# Dynamic protein structures in solution: decoding the amide I band with 2D-IR spectral libraries and machine learning

**DOI:** 10.1039/d5sc09973k

**Published:** 2026-01-08

**Authors:** Amy L. Farmer, Kelly Brown, Sophie E. T. Kendall-Price, Partha Malakar, Gregory M. Greetham, Neil T. Hunt

**Affiliations:** a Department of Chemistry and York Biomedical Research Institute, University of York York UK neil.hunt@york.ac.uk; b STFC Central Laser Facility, Research Complex at Harwell, Harwell Science and Innovation Campus Didcot UK

## Abstract

The dynamic three-dimensional structures of proteins dictate their function, but accessing structures in solution at physiological temperatures is challenging. Ultrafast 2D-IR spectroscopy of the protein amide I band produces a spectral fingerprint that derives directly from the 3D backbone structure within minutes, using microlitres of label-free samples, in aqueous (H_2_O) solution and with picosecond time resolution. However, transforming 2D-IR fingerprints into quantitative, solution-phase protein structures relies on decoding the fundamental link between the atomistic structure and the 2D spectrum. We demonstrate a top-down approach to solution-phase protein structure determination that combines 2D-IR spectral libraries with machine learning (ML). Using a dataset consisting of 6732 spectra of 35 proteins in H_2_O that span a range of structures, Support-Vector Machine (SVM) models classified unknown protein samples according to structural content and measured quantities of α-helix and β-sheet with an RMS error of ≤7%. The potential for hybrid 2D-IR-ML tools to predict the number and length of helices in a protein, and identify the presence of parallel and antiparallel β-sheets from the 2D-IR fingerprint is also demonstrated. These results lay the groundwork for rapid, quantitative analysis of dynamic protein structures under physiologically relevant conditions.

## Abstract 2

Ultrafast 2D-IR spectroscopy and machine learning combine to determine label-free protein secondary structures in solution.

## Graphical abstract

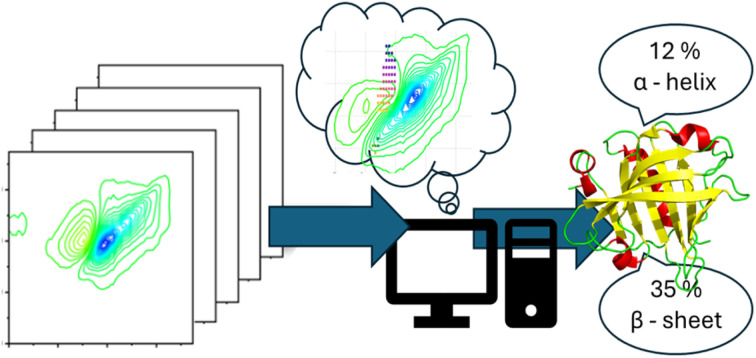

## Introduction

The link between structure and function means that measuring a protein's structure is crucial to understanding and influencing its behaviour. Powerful methods such as cryo-electron microscopy (cryo-EM) and X-ray diffraction provide atomistic structures that have transformed our understanding of biological molecules.^1^ However, measuring structures at biological temperatures in solution, where structural dynamics can dominate, remains a significant challenge. NMR spectroscopy can deliver detailed solution-phase protein structures with dynamic insight but requires labelled or enriched proteins.^2^ Circular dichroism (CD) spectroscopy offers label-free solution phase secondary structure information, but its accuracy when used for absolute quantification has been shown to vary, particularly in systems with mixed α-helix and β-sheet content.^3–6^ This leaves an opening for complementary techniques that offer fast, quantitative, label-free insight into protein structures in solution. Such methods could be valuable in accessing protein dynamics and in guiding the use of more time and resource intensive tools.

Since its first demonstration,^7^ ultrafast two-dimensional IR (2D-IR) spectroscopy has been used to probe protein structure. The protein amide I vibrational mode, essentially of the C=O stretch of the peptide unit, is extremely sensitive to the three-dimensional conformation of the folded peptide chain. Inter-residue vibrational coupling, hydrogen bonding, solvation, structural dynamics, and the local electrostatic environment all contribute to the form of the amide I absorption band.^7–17^ By spreading the amide I signature over two frequency axes, 2D-IR measures a detailed fingerprint that is directly linked to the unique structure of the protein, while picosecond time resolution offers dynamic insight. Furthermore, 2D-IR suppresses the background H_2_O signal that inhibits linear absorption spectroscopy, enabling measurements under more physiologically relevant conditions.^13^ Changes in protein structure and dynamics upon drug or ligand binding and melting have been measured with 2D-IR,^18–24^ as have proteins in complex biological matrices such as blood serum, including clinical samples.^18,19,25^ Progress in 2D-IR experimental methods has reduced spectral acquisition times to just a few minutes, while data pre-processing has enabled accurate standardisation of spectra from different samples.^26–28^

This combination of bond-level resolution and sensitivity to small changes in label-free molecular structure and dynamics means that 2D-IR offers a promising route to accurate protein structure determination in solution. But decoding the structure–spectrum relationship that links the 3D arrangement of atoms to the spectral fingerprint remains a barrier to quantitative interpretation of the spectra. Bottom-up experimental methods using bespoke peptides, small model proteins or isotopic labelling have allowed specific regions of the amide I band to be identified with certain types of structural elements.^7–9,14,17,26^ Transition dipole strength analysis has also been used to examine protein secondary structure,^15,16,29,30^ including to determine the maximum α-helical length in a protein.^30^ Singular value decomposition (SVD) was used to quantify protein structure from 2D-IR spectra of proteins in D_2_O in 2011,^31^ achieving accuracies approaching those of CD, but was not pursued further. Simulations based on molecular dynamics and frequency maps are also instructive,^32–37^ but challenges arise from computational cost and a lack of experimental data for validation.^38^

Here, we describe an alternate, top-down, approach that combines the spectral information density of 2D-IR with the strengths in pattern recognition of machine learning (ML). We have created the first label-free 2D-IR protein spectral library in H_2_O-based buffer solutions, containing 6732 spectra of 35 different proteins. The proteins were selected to encompass a range of secondary structure configurations, and each have a high-resolution structural analysis available *via* the protein data bank (PDB).^39^ Our goal, inspired by ML applications to simulated spectral datasets^40–42^ and multidimensional NMR,^43^ was to use ML firstly to classify protein spectra according to structural type, and then to quantify secondary structure content. We show that both aims are achievable, and that 2D-IR-ML has the potential to go further and predict the number of helices present in protein structure and identify the presence of parallel and antiparallel β-sheets. Based on this, we consider the scope for 2D-IR to play a meaningful future role in determining dynamic protein structures in solution.

## Experimental

### Protein selection and sample preparation

Thirty-five proteins were selected from the cSP92 library of commercially available proteins.^39^ All proteins were purchased from Merck and used without further purification. Each was available as a high-purity lyophilized powder with a high-resolution X-ray crystal structure published in the PDB. Selections were made to provide a broad sample of secondary structural space, spanning 0–71% α-helix and 0–48% β-sheet (Table S1). The proteins were dissolved in a pH 7.4 aqueous (H_2_O) phosphate buffer at a concentration of 30 mg mL^−1^, giving, as near as possible, a consistent concentration of amino acid residues between samples. This was chosen over molar concentration because of the spread of molecular weights across the proteins. For 2D-IR measurements, 15 µL of each protein solution was placed between two CaF_2_ windows in a standard transmission cell. For each sample, the absorbance of the *δ*_OH_ + *ν*_libr_ combination mode of H_2_O centred at 2130 cm^−1^ was ∼0.1, corresponding to a path length of ∼2.75 µm.^13^

### 2D-IR spectroscopy

The 2D-IR spectra were recorded on the LIFEtime instrument at the STFC Central Laser Facility.^44^ The pump and probe pulses were centred at 1650 cm^−1^ with a pulse repetition rate of 100 kHz and bandwidths of ∼80 and ∼200 cm^−1^ respectively. The coherence time (*τ*) between the two pump pulses was scanned from 0 to 3 ps with a 24 fs step size using a pulse shaper. The 2D-IR signal was detected using two 128-element mercury-cadmium-telluride (MCT) detectors, giving a probe frequency axis resolution of ∼3 cm^−1^. For each sample, spectra were recorded using parallel pump-probe polarization and measured three times (cycles). Eleven waiting times (*T*_w_) were measured between 250 and 300 fs in 5 fs steps. These *T*_w_ values were selected to maximise the number of spectra available for ML by introducing variability of signal size and noise into the spectra while avoiding too great a *T*_w_ dependence on the size and shape of the signal due to vibrational relaxation.^13^ Each protein sample was measured in triplicate in different transmission cells, except for peroxidase and prealbumin which were measured twice and once, respectively due to limited supply (<1 mg). Each measurement cycle was treated as an individual spectrum, giving 198 spectra each for 33 of the proteins (3 cycles, 11*T*_w_ values, and 2 detectors, in triplicate), 132 for peroxidase, and 66 for prealbumin; a total of 6732 spectra.

### Formatting the 2D-IR library

**Fig. 1:**
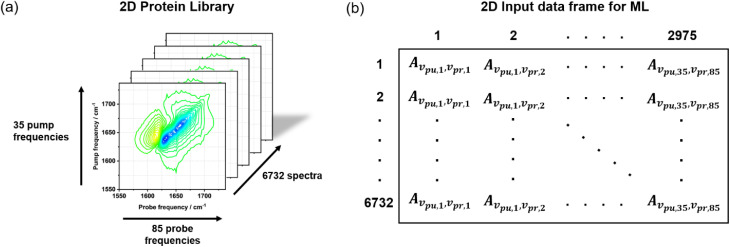
Schematic diagram outlining how the protein library (a) was formatted into an input data frame for ML analysis (b).

The amide I response in each spectrum was isolated by extracting the region between 1550–1737 cm^−1^ of both the pump and probe axes. This resulted in 35 pump frequencies and 85 probe frequencies, a total of 2975 points per spectrum (Fig. 1(a)). For the 2D protein library, pump slices were concatenated such that each 2D-IR spectrum was represented by a 2975-dimensional vector. Each vector was then combined into an N × M data frame comprising N spectra and M features, where each feature is the spectral amplitude, A, at each point on the 2D-IR spectrum (Fig. 1(b)). As the probe axis was recalibrated on each day of data acquisition, there were small discrepancies between the wavenumber assigned to each probe pixel (±1.5 cm^−1^, lower than the spectral resolution). This was accounted for by binning the data according to the average of the wavenumber spread.

Each spectrum was labelled with either a class assignment, the proportions of α-helix and β-sheet, the number of α-helices, or the proportions of parallel and antiparallel β-sheet (Table S1) depending on the aim of the ML task (see below). Protein structural properties were determined through the Dictionary of Protein Secondary Structure (DSSP) algorithm.^45^

### Machine learning

For ML analysis the data library was separated into independent training and testing sets. During training, a model will learn any relationships between the training spectra and their labels, and how to optimise its parameters to accurately predict the label of an unseen test spectrum. Keeping the test and training datasets separate is essential to measure generalizability, that is, how well the model can be applied to unseen data.

To avoid leaking testing data into the training process, we used a nested-cross validation framework (nested-CV, Fig. S1). This contains an inner-loop CV, where the model's parameters are tuned according to the training data, from a given iteration of the outer-loop CV, where the model's final performance is tested. This process is repeated for all folds of the outer-loop CV. As the library contains multiple spectra of 35 proteins, a group CV was used for both loops where each group contained all of the spectra of one protein (35 groups in total).

The inner-loop consisted of a pipeline within a 5-fold group CV containing two transformers and a final predictor. The first transformer was a standard scaler that standardised the features to a normal distribution of zero mean and unit variance. The second was a feature selection module where the most relevant features for accurate learning were identified. This was used to minimise the risk of overfitting and improve model performance. The feature selection method and final predictor were varied according to the task and these are outlined below. All of the models were implemented through a custom python script using the scikit-learn package.^46^

#### Classification

For classifying protein spectra according to structural type, the input data frame was labelled with the appropriate protein structural class assignments (see below and Table S1) and passed through the nested-CV. In the inner-loop, two different feature selection methods (Principal Component Analysis (PCA) and an ANOVA-*F* test) were assessed. For PCA-based feature selection, the principal components (PCs) were used as input for the final predictor, whilst for the ANOVA-*F*-test the features (spectrum pixels) with the largest *F*-values were used. The number of PCs and features to input were selected during the hyperparameter tuning process in the inner loop.

Four different final predictors were tested: Support Vector Machine (SVM) with a radial basis function (RBF) kernel, k-Nearest Neighbours (kNN), Decision Tree (DT), and Random Forest (RF). The training: testing split of the outer-loop was varied, where the performance of three randomly generated 80 : 20 (training : testing) splits and a Leave-One-Out Cross Validation (LOO-CV) were examined. For the LOO-CV, the total protein library was separated into the 35 spectral groups and for each outer-loop iteration a different spectral group was used as the test set. As ANOVA-*F* feature selection with the SVM predictor was found to deliver the best performance for classification (see Results), these were taken forward to subsequent tasks.

#### Secondary structure quantification

The input data frame was labelled with appropriate secondary structure data, such as the α-helix and β-sheet proportions, calculated using the DSSP (Table S1). SVM for regression was used as the final predictor. To distinguish between classification and regression pipelines, the acronyms Support Vector Machine for Classification (SVC) and Support Vector Machine for Regression (SVR) will be used. Further details are presented in the Results section.

#### ML assessment

The performance of the ML model was assessed for the classification tasks using the accuracy (percentage of correct predictions), Cohen's Kappa, precision, recall and *F*_1_ score parameters, and for the regression tasks using the pooled standard deviation (*S*_pooled_) and root mean squared error (RMSE) parameters. The RMSE parameter was used to measure the difference between the DSSP calculated secondary structure proportion and the predicted proportion, whilst the *S*_pooled_ parameter was used to measure the spread in prediction across the repeated spectra of each protein, giving an indication of the tolerance of the ML method to experimental differences.

## Results

### 2D-IR spectra: protein library

**Fig. 2:**
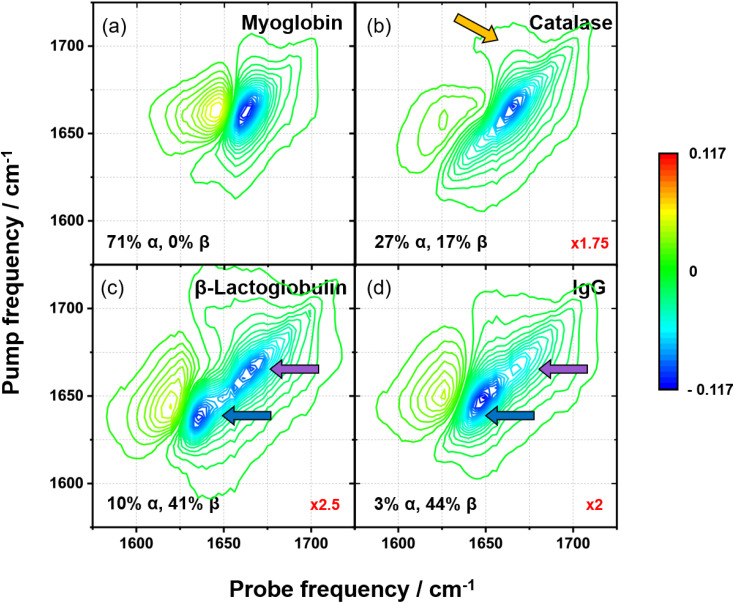
Amide I 2D-IR spectra of (a) myoglobin, (b) catalase, (c) β-lactoglobulin, and (d) IgG. Quoted on each spectrum are the proportions of α-helix and β-sheet calculated from the DSSP. The red numbers in the bottom right corners of each panel show the magnification factors used to plot the spectra on the same scale.

Sample 2D-IR spectra of four proteins selected from the library are shown in Fig. 2. Exemplar spectra of the remaining proteins are given in the SI (Fig. S2). Each spectrum (Fig. 2) contains a negative feature (blue) on the spectrum diagonal and a positive feature (yellow/red) shifted to lower probe frequency. These are assigned to the *v* = 0 → 1 and *v* = 1 → 2 transitions of the amide I vibrational mode of the protein, respectively.

It is well established that an α-helix structure gives rise to an amide I response that features two contributions; an intense A-symmetry mode centred at ∼1650 cm^−1^ and a much weaker, rarely-resolved, E-symmetry mode near ∼1640 cm^−1^.^9,29,47^ Similarly, the inter-peptide coupling in β-sheets leads to the presence of an intense *v*_⊥_ mode near 1630–1640 cm^−1^ and a weaker *v*_‖_ mode near 1670–1680 cm^−1^.^14,15,17,47,48^ As a result, the 2D-IR response of a β-sheet manifests as a ‘z-shaped’ pattern where the *v*_⊥_ and *v*_‖_ diagonal peaks are linked by off-diagonal features arising from the coupling between them. The *v*_⊥_ mode is known to shift to lower frequencies and increase in amplitude with increasing length of the β-sheet.^14,17^ Based on this information, it is possible to account qualitatively for some of the results in Fig. 2. The *v* = 0 → 1 transition of the amide I band of myoglobin has one main feature centred at 1660 cm^−1^ (Fig. 2(a)) that derives from its almost entirely α-helical structure, accounting for the expected upshift of ∼10 cm^−1^ in H_2_O-based solvents relative to the more normally used D_2_O.^49^ In the case of catalase, an elongation of the amide I band along the spectrum diagonal and more apparent off-diagonal features (*e.g.* pump, probe = 1705, 1655 cm^−1^, orange arrow in Fig. 2(b)) are observed due to the greater β-sheet content.^50^ Both IgG and β-lactoglobulin have structures that are dominated by β-sheets, and the ‘z-shaped’ pattern with significant *v*_⊥_ mode amplitude (pump = probe = 1649 and 1638 cm^−1^, respectively, blue arrows, Fig. 2(c and d)) is apparent in both cases. However, despite both proteins having similar proportions of α-helix and β-sheet (Fig. 2(c, d) and Table S1),^51,52^ the 2D-IR responses differ in the intensities of the 1660 cm^−1^ diagonal contributions (purple arrows) and the ratio of the amplitude of this feature to that of the *v*_⊥_ peak (blue arrows). This situation exemplifies both the sensitivity of the 2D-IR amide I response and the challenge faced by structure quantification methods as a given secondary structure content does not necessarily produce one distinct spectral pattern. This is due to the influence of other factors such as chain length, the strength of the vibrational coupling, and the global environment of the structural feature including tertiary structures.^15,53^ We therefore explore whether ML-based approaches offer a route towards unravelling this nuanced spectral fingerprint.

### Classifying secondary structure

#### Classification performance

We start by using ML analysis to classify protein 2D-IR spectra into defined categories according to their structural composition, as might be applied in protein quality control and regulatory analysis.

**Fig. 3:**
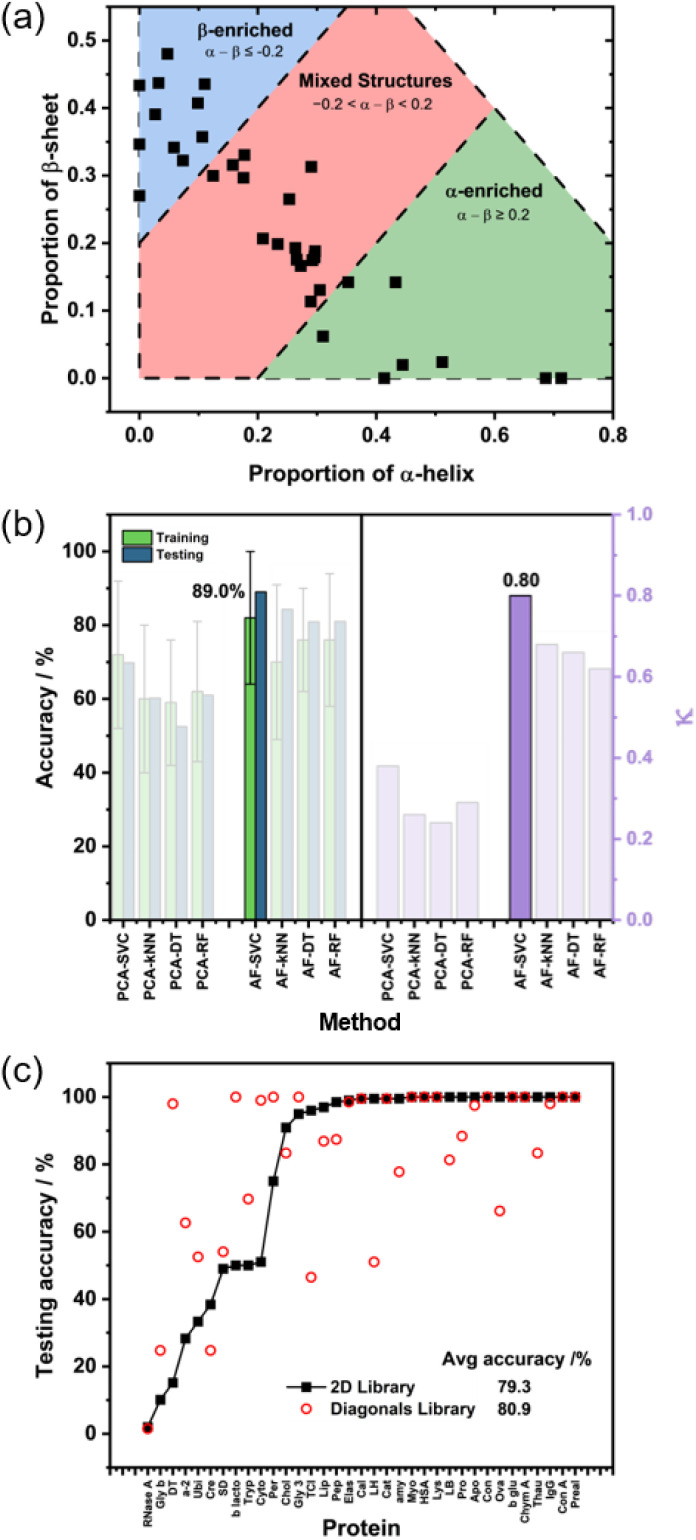
(a) The proportions of α-helix and β-sheet calculated from the DSSP of the 35 proteins in the protein library. The blue, red and green regions indicate which proteins are grouped into the ‘β-enriched’, ‘mixed structures’, and ‘α-enriched’ classes, respectively (b) (left panel) The average training accuracies across a 10-fold CV and testing accuracies, and (right panel) the Cohen's kappa values for the 8 methods trained on the 2D library. The best performing method, AF-SVC, has been highlighted in both panels. (c) The testing accuracies for each of the proteins from an outer-loop LOO analysis using the AF-SVC model trained on the 2D library (black squares) and the PCA-SVC model trained on the diagonal library (red circles). The proteins have been ordered on the *x*-axis by increasing testing accuracy from the 2D results. The inset gives the average testing accuracy across the 35 proteins for each library.

The proteins in the library were assigned to one of three classes, ‘α-enriched’, ‘β-enriched’ and ‘mixed structure’ (Fig. 3(a)) according to the definitions:

(1) α-Enriched: *α* − *β* ≥ 0.2,

(2) β-Enriched: *α* − *β* ≤ 0.2,

(3) Mixed structure: all other proteins.

Where *α* and *β* represent the fraction of α-helix and β-sheet respectively. This resulted in eight proteins (1518 spectra) in the ‘α-enriched’ class, 16 (3168) in the ‘mixed’ class, and 11 (2046) in the ‘β-enriched’ class.

The aim was to train an ML model to assign the correct structural class to an unseen (test) protein or group of proteins based on their 2D-IR spectra. A variety of feature selection methods and final predictor combinations were assessed. The best performance across three randomly generated 80 : 20 (training : testing) splits was delivered by an SVC model combined with the ANOVA-F (AF) feature selection. This combination performed well according to a number of metrics (Fig. 3(b) and Table S2), not least showing strong predictive power with a testing accuracy of 89% and a *κ* value of 0.80. The F1 scores between the 3 classes were also generally well balanced, with the α-enriched class giving the smallest score (0.80 *vs.* 0.90 for the other classes, Table S2). A breakdown of the individual performances for the three test sets using the AF-SVC model are given in the SI (Fig. S3 and Table S3).

To analyse the performance of the AF-SVC model further, an outer-loop Leave-One-Out analysis (LOO) was implemented in which the spectra of each protein in turn were used as a test set and the remaining library as the training set. This is useful in giving a better estimate of model performance, especially when the data set is relatively small.^54^ The results (Fig. 3(c)) show that the average testing accuracy across the 35 proteins was 79.3%, with the model achieving ≥95% accuracy for 23 proteins.

#### Spectral region selection

Given the success of the classification model at identifying protein fingerprints according to structural type, it is instructive to examine the regions of the 2D-IR spectrum that the model used as a basis for classification. The ANOVA-*F* test, used for feature selection, identifies the pixels in the spectra with the highest statistical significance (*F*-value). The number of pixels used by the SVC model for predictions was decided during hyperparameter tuning in the inner-loop of the nested-CV. In each case, 50 pixels were identified.

**Fig. 4:**
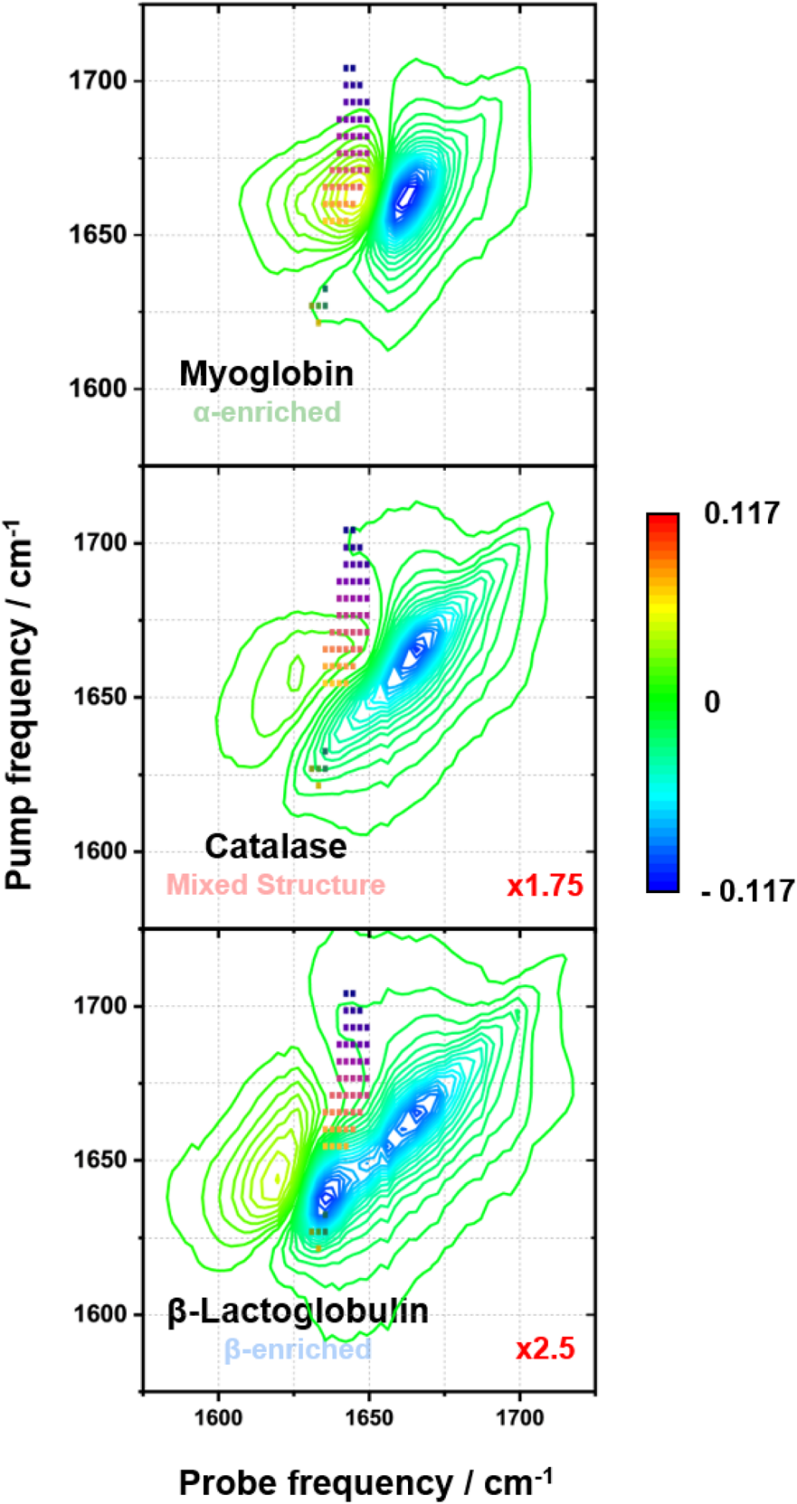
The positions of the 50 features with the highest *F*-values from an ANOVA-*F* test on the total protein library (squares) represented on one example 2D-IR spectrum from each class (myoglobin for α-enriched, catalase for mixed structures, and β-lactoglobulin for β-enriched). The red numbers are the magnification factors used to set the spectra to the same *z*-axis.

Fig. 4 shows the 50 features with the highest *F*-values from an ANOVA-*F* test on the total protein library (coloured squares) overlaid with the 2D-IR spectrum of one example from each protein class (myoglobin: α-enriched; catalase: mixed and β-lactoglobulin: β-enriched). It should be noted that, in the reported implementation of the AF-SVC model, an ANOVA-*F* test was performed on the given training set, not the total library. As this is a supervised feature selection method, this means that the features with the highest *F*-values will vary for each unique training set. However, inspection of the results of an ANOVA-*F* test across the training sets used and the total protein library showed that, while some of the specific selected features varied, the features lay consistently in this same region of the spectrum.

**Fig. 5:**
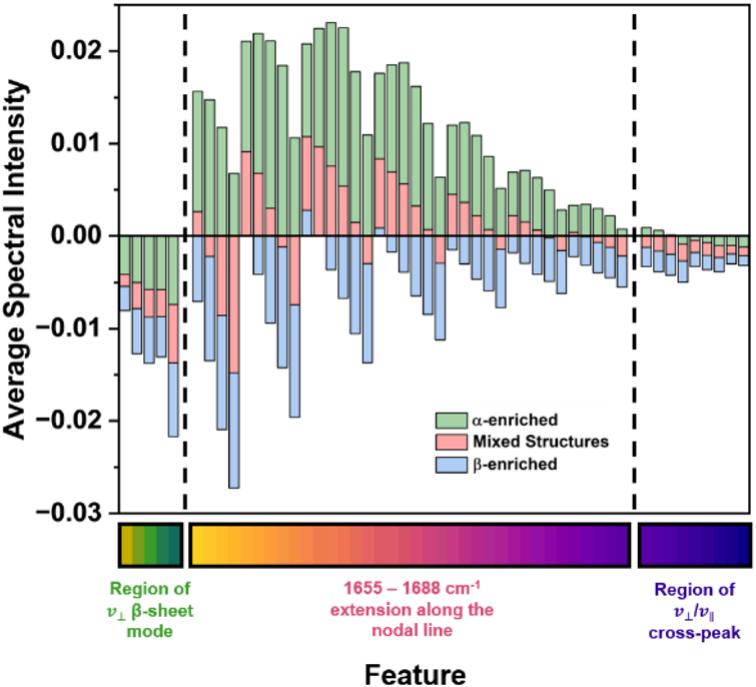
A bar graph showing the average spectral amplitude at the 50 selected features for the three classes: α-enriched; green bars, mixed structures; red bars, and β-enriched; blue bars.

The selected features track the change in position and shape of the nodal line between the negative *v* = 0 → 1 and positive *v* = 1 → 2 peaks across the three classes. The appearance of a high frequency cross peak and the edge of the *v*_⊥_ β-sheet mode are also used as spectroscopic markers. Across the different protein classes, the selected features exhibit a change in amplitude from positive (myoglobin, α-enriched), through to near zero (catalase, mixed), and to negative (β-lactoglobulin, β-enriched) (Fig. 5). This change in the position and shape of the nodal plane can be explained by the complex overlap of positive and negative features that occurs when several structurally unique amide I contributions are present. This is especially true when there are cross peaks that lie close enough in frequency and have a large enough bandwidth to interfere with the *v* = 0 → 1 and *v* = 1 → 2 bands as this can lead to further convolution of the response. From this, we conclude that the model is learning to distinguish between the classes successfully based on the spectral amplitude in the regions known to be associated with the presence of β-sheet structures.^17^

#### The 2D-IR spectrum diagonal

From the perspective of the traditional spectroscopist, *i.e.* without the application of ML strategies, it is interesting that the model selects a more complex portion of the amide I response than a conventional amide I analysis might use.^16,55^ The link between the 2D-IR diagonal and the amide I absorption band, as measured in 1D spectroscopy, would tend to lead to analyses based on the features appearing along the pump = probe frequency line. Although not selected by the freely applied ML method, it is instructive to compare the result with a model that is directed to focus upon the diagonal, effectively applying human-in-the-loop or domain knowledge.

In this case, the input data frame consisted of the diagonal slices through the 2D-IR spectra (35 data pixels) annotated in the same way as for the full 2D-IR model described above (Fig. S4). PCA and ANOVA-*F* feature selection was applied alongside SVC, kNN, DT and RF predictors. PCA-SVC was found to perform best, reaching a promising 85.8% accuracy though with a lower *κ* value of 0.66 across the three randomly generated 80 : 20 splits (Fig. S5). Examining the results of the LOO analysis (Fig. 3(c)) shows that, although a number of proteins were classified 100% correctly, there is a greater spread of misclassifications than when using the full 2D-IR plot.

### Protein secondary structure quantification with 2D-IR-ML

In this second application, we report the ability of ML to quantify secondary structure content from 2D-IR spectra. The aim here was to determine the proportions of α-helix and β-sheet in the protein structure from its 2D-IR spectrum. For this task, the input data frame was labelled with α-helix and β-sheet proportions calculated from crystallographic data *via* the DSSP (Table S1). As the AF-SVC model performed best in the classification task, the same model was used here but with the analogous regression function (AF-SVR).

To account for the negative correlation between the proportions of α-helix and β-sheet structures in the protein library (Fig. 3(a) and Table S1), a regression chain was used.^56^ Here, the training set is passed first into an ML model which performs a prediction of β-sheet content, from which the β-sheet predictions and training set are then run through a second ML model which generates a prediction of α-helix content. This specific order was chosen as the selected features for ML analysis cover more regions known to be associated with the presence of β-sheet structures, though we found little difference in performance when the chain order was reversed.

As the ANOVA-*F*-feature selection test only measures the statistical significance of the features of the spectrum against one target variable, the implementation had to be adapted for use with a regression chain. Two independent *F*-tests were performed, one using the proportions of α-helix as a target variable, and the other using the proportions of β-sheet. The highest scoring features from both tests were then combined into one list and the duplicates removed to give a final list of features that were used as input for both ML steps. This also meant that the optimal number of features had to be identified manually outside of the inner-loop CV. For this, the number of features selected from the separate *F*-tests for combination into a final list were varied, and each final list passed into a pipeline without a feature selection transformer for hyperparameter tuning. The final list that gave the smallest training RMSE was selected to produce a tuned model for assessment against the test set of the outer-loop. 40 features from both independent *F*-tests were consistently selected, giving 47 features in total. These are represented in Fig. S6.

**Fig. 6:**
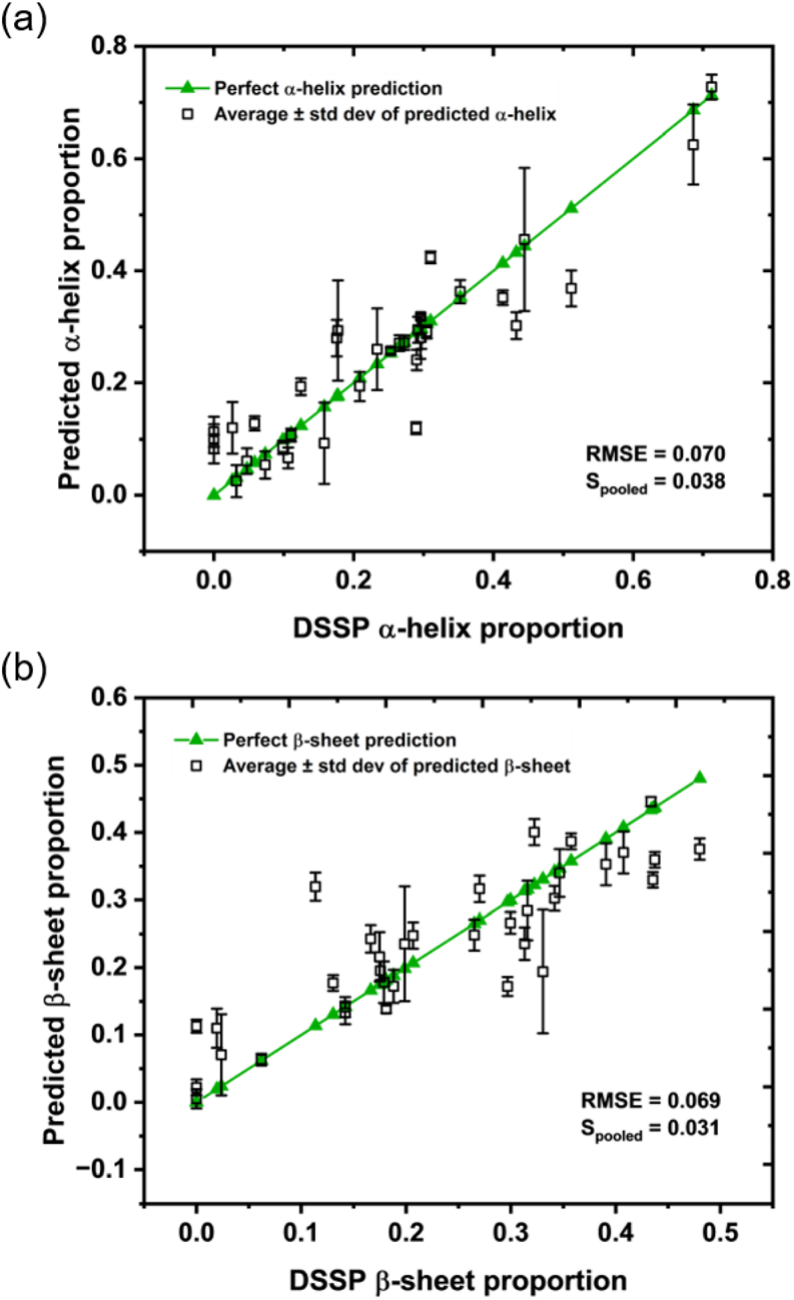
The predicted (a) α-helix and (b) β-sheet proportions from a LOO analysis using the AF-SVR pipeline trained on the 2D library, where each open black square represents the average predicted proportion across the repeat spectra in each protein group. In each panel, the green line represents a perfect prediction where the green triangles are positioned at the DSSP calculated proportions of α-helix and β-sheet for the 35 proteins.

First, the performance of the AF-SVR approach was assessed using an outer-loop LOO analysis where, after training, the model was tested against a given protein spectral group. The results of this are shown in Fig. 6 where each black square represents the average predicted secondary structure proportion over all of the spectra in the protein spectral group for each protein ((a) and (b) show α-helix and β-sheet results respectively). Error bars show the standard deviation of the predicted values. A perfect prediction is shown in green. A RMSE in prediction of ≤7% for both α-helix and β-sheet content shows that the AF-SVR model is predicting the secondary structure distribution well, while the linearity also displays good agreement across the range of structures included in the library. When RNase A and DT diaphorase, two of the six proteins that the model produced some of the lowest testing accuracies for in the structural classification task, were removed, these RMSE values reduced to 6.2% and 5.6% for α-helix and β-sheet, respectively.

Despite this demonstration of good predictive power for the SVR approach, there is still error between the predicted and actual secondary structure proportions. It is clearly plausible that a larger protein library that covers a more diverse structural space would lead to improved predictions. However, it must also be considered that, since the model is examining solution-phase spectra labelled with DSSP calculated crystallographic secondary structure proportions, some error might arise from any discrepancies between the crystal structure and the dynamic structure that exists in solution. Alternative sources of structural information may therefore also need to be considered for the training process.

#### The 2D-IR spectrum diagonal

Once again, we compared the result with a model directed to use the spectrum diagonal. In this case, the best-performing PCA-SVR model was carried forward from the classification task. The results showed that the diagonal portion of the spectrum also delivers information relating to the secondary structure, with similar prediction accuracies of around 6.5% for α-helix and β-sheet, though a slightly poorer *S*_pooled_ than when using the full 2D-IR spectrum (Fig. S7). Examining the form of the PCs used by the model (Fig. S8) also shows fewer features that could be directly attributed to secondary structure elements using prior knowledge of the amide I band.

### Predicting other structural properties

After exploring classification and regression approaches to determining α-helix and β-sheet content, we move to assessing whether ML analysis of the protein library could be used to predict other structural properties. The aim is to survey the potential for a larger library to go significantly beyond the ability to measure secondary structure, with an outlook towards using 2D-IR for detailed evaluation of protein structures in H_2_O.

The length of an α-helix is known to alter a protein's IR response. Longer helices typically shift the A-mode to lower frequencies, whilst the degeneracy of the E-mode is lost in shorter helices.^47,57^ Consequently, helices shorter than around six residues can generate unusual responses, with a number of bands distributed throughout the amide I region.^58^ Therefore, subtle differences in the 2D-IR spectra of proteins containing long or short α-helices would be expected.

Taking this into consideration, we attempted to further classify the proteins in the protein library using ML according to the length of the α-helices they contain. The proteins were separated into two classes; ‘short helices’ if they contained no α-helices longer than 15 residues, and ‘long helices’ if they contained α-helices longer than 15 residues. The cut-off value of 15 residues was selected to give an even distribution of proteins between the two classes (16 ‘short helices’ and 19 ‘long helices’). This classification also ensured the separation of proteins containing extremely long helices (*e.g.* glycogen phosphorylase b contains a 30-residue long α-helix, and human serum albumin a 33-residue long α-helix).

An ANOVA-*F* test was again used as the feature selection method, and assessed in combination with SVC, kNN and DT across three randomly generated 80 : 20 (training : testing) splits. All three of these models produced good testing accuracies but poor *κ* values (72% and 0.413 for SVC, 70% and 0.370 for kNN, and 74% and 0.489 for DT), and so an ensemble approach, Adaptive Boosting (AdaBoost), was considered. AdaBoost is a common boosting algorithm that assembles a collection of weak learners, usually single-level decision trees, into one larger classifier.^59,60^ At each iteration, the training examples are re-weighted such that each subsequent weak learner focuses on correcting the misclassified samples from the previous weak learner. These ensemble methods can better handle outliers and limit the chance of overfitting. On average across the three test sets, an AF-AdaBoost pipeline delivered an 83% testing accuracy, with a more confident *κ* value of 0.623. These predictions were made using the same region of the amide I response as the previous classification and regression models (see Fig. S9), which is a region that would be altered by the described moving A-mode and splitting E-mode with helix length.

Predicting the number of α-helices in a protein was also attempted. Using an independent AF-SVR model, a RMSE across 6 randomly selected proteins of 8.6% was obtained. This reduced to 4.8% when a regression chain was used that predicted α-helix proportion first which then fed into a prediction of the number of α-helices. The prediction was therefore improved by taking into account the weak positive correlation between the total proportion and number of α-helices in a protein structure. It would be important to also consider the total number of residues in a protein in this context (a small protein could have a large proportion of α-helix but not necessarily a large number of helices) but it was not possible to predict this well using the protein library 2D-IR spectra alone.

The β-sheet properties of a protein were also expanded upon through a prediction of the proportions of parallel and antiparallel β-sheet. Again, using independent AF-SVR models, a RMSE across 6 randomly selected proteins of 4.7% was obtained for antiparallel sheets. A much larger error of 8.8% was obtained for parallel sheets which, when considering that the proportions of parallel sheet in the library only vary between 0 and 13.1%, is comparatively very poor. By examining the features of the 2D-IR spectrum selected during the *F*-test for parallel sheets (Fig. S10(a)), the reason for this performance becomes clear. It is the high probe frequency region just outside of the amide I response that is selected, where any model trained on these features would likely overfit to the noise and incorrectly use that as a marker of parallel sheets. This is possibly a consequence of the little variance in the proportions of parallel sheet across the library (0 to 13.1%) where 14 proteins (almost half of the dataset) have 0% parallel sheet. In contrast, the *F*-test for antiparallel sheets selects the familiar nodal region (see Fig. S10(b)). When the SVR model for parallel sheets is made to also consider these antiparallel sheet selected features, the error reduces to 4.2%. This emphasises the necessity of domain knowledge for ML applications, especially when operating at the limit of what our protein library can achieve.

Overall, these predictions of other structural properties further confirm the potential of 2D-IR-ML methods for protein structural analysis. Whilst it is clear that more work, and more spectral data on a wide range of proteins, is necessary to improve predictive capabilities, this provides a proof-of-concept for the ability of ML analysis to retrieve the dense structural information from 2D-IR spectroscopy.

## Discussion

The ability of the ML models to classify proteins according to secondary structure and to predict the secondary structure content based upon label-free spectra, acquired rapidly in the solution phase, establishes proof-of-concept for a number of applications combining 2D-IR and ML for protein analysis. These range from quantitative monitoring of structural changes in response to chemical or thermal conditions, to practical applications such as the comparison of structures of biosimilar molecules to those of a biological target, as is required in therapeutic regulation. Applications to the drug design pathway can also be envisaged by probing changes in structure or protein dynamics caused by drug binding. Such future applications will motivate further development of these library-based tools.

It is constructive to compare the results of this study to the state-of-the art. An error in prediction of ≤7% for both α-helix and β-sheet obtained here compares favourably with the only other report of using experimental 2D-IR spectra for ML-based quantitative secondary structure prediction, which used singular value decomposition (SVD) on a library of 16 proteins measured in D_2_O.^31^ This study assumed that a total protein 2D-IR spectrum can be made by the linear addition of contributions from pure α-helix, β-sheet and ‘unassigned structures’. While the approach was successful, producing RMSE values of 12.5 and 9.2% for α-helix and β-sheet, respectively,^31^ the method reported here produces more accurate results. Given that the new data is obtained in the more physiologically relevant H_2_O, where data collection is more challenging, this indicates that the ML approach is benefitting from the greater amount of experimental data that we are able to include here.

The performance metrics of our model also compare favourably to those from Circular Dichroism (CD) spectra. For an example SVM model, RMS errors of 5.7 and 6.9% for α-helix and β-sheet, respectively, were obtained.^3^ In the CD study, the model was trained on a larger database of 72 proteins (SP175 reference set), of which there is a match in 21 proteins with this library, but only in six PDB structures.^61^ So, whilst the performance of the model reported here is numerically poorer than the example CD trained model, it emphasises the prospect of ML protein analysis in tandem with a 2D-IR spectral library, as performance is still good even when trained on a dataset with comparatively lower structural diversity. As such, this provides a vindication for the further development of 2D-IR ML models but also reinforces the importance of library size. The demonstrated ability of 2D-IR to go beyond α-helix proportion to identify size and number of helix units and predict proportions of parallel and antiparallel β-sheets also shows considerable potential for future development beyond basic secondary structure elements.

It is particularly interesting that the outcome of our ANOVA-*F* feature selection process led to the ML models exploiting the off-diagonal region of the 2D-IR spectrum, rather than the more traditional spectrum diagonal. Indeed, a direct comparison here using the diagonal slice has shown that, while the latter contains sufficient information to make a good prediction, using the full spectrum leads to a more interpretable, and for most metrics, better outcome. This conclusion has also been reached in previous studies employing simulated 2D data and machine learning models, where it was found that the off-diagonal region of the spectrum was important for improved model performance.^41,42^ Analogous observations were also made recently when applying 2D-IR-ML to examine the spectra of biofluids.^25^ As well as validating the use of ML, this provides an important point of distinction with methods such as CD, which use a linear, one-dimensional spectrum to achieve the same quantification. The ability to unravel complex spectral contributions *via* a second spectral dimension and probe the inter-peptide interactions occurring within the structure provide a basis for a more detailed quantitative analysis of 2D-IR spectra than is currently possible.

As with all ML-based approaches, it will be important to keep adding to the protein spectral library in order to enhance its predictive ability. This will bring challenges in terms of transferring data between instruments and protein conditions, though with sufficient data and careful standardisation, these hurdles should be surmountable. There is also considerable scope to combine simulated and experimental data to expand models, improve simulations, and allow access to prediction of further structural properties, leading towards a detailed understanding of the structure-spectrum relationship.

Whilst the work here represents another step towards potential 2D-IR structural analysis tools, there are practical factors that must be considered. First is the sensitivity of 2D-IR, which can reduce the concentration range over which the technique can be applied. Using current technology, the detection limit for a protein in H_2_O is ∼5 mg mL^−1^, or around 70 µM for a protein like Human Serum Albumin (HSA). This is comparable to, and in many cases lower than, the concentrations used for NMR or to prepare samples for crystallography and cryo-EM. The value of 2D-IR is enhanced by the fact that the proteins are both unlabelled and fully solvated, which is not the case for many other methods. At higher concentrations, protein aggregation can be an issue, but we find that for the test protein BSA at three different concentrations, there is no indication of aggregation (Fig. S11). More broadly, we also note that when aggregation occurs it is clearly identifiable by a characteristic signature near 1620 cm^−1^.^62,63^ Separate to aggregation, the ability of 2D-IR to detect the formation of protein multimers in solution is an important question that will need to be addressed. Overall, while there will inevitably be proteins that cannot be studied in solution due to a lack of solubility, this issue is likely to be no more prohibitive than, for example, proteins that do not crystallise well. A multi-method approach will always be vital to build a picture of a protein's structure and dynamics.

A second issue relates to the instrumentation. At present, 2D-IR is a somewhat niche technique, relying on ultrafast lasers and specialist laboratories. However, if one considers that since the first measurements in 1998,^7^ laser systems have advanced to robust turn-key sources and that one-box spectrometers have become commercially available, then the direction of progress towards an accessible solution becomes clear. By comparison, 28 years elapsed between the characterisation of nuclear magnetic moments and the first commercial NMR spectrometer, and a further 17 years before Fourier Transform technology emerged.^64^ Similar timescales were also required for techniques like cryo-EM to reach their full capabilities. It is therefore reasonable to assume that the accessibility and applications of 2D-IR will only continue to progress.

## Conclusions

We have shown that combining 2D-IR spectroscopy of the amide I fingerprint of a library of proteins with machine learning analyses gives a route to label free quantification of structures in solution. We highlight two applications, using 2D-IR for structure classification, and for direct quantitative analysis of secondary structure. Both approaches achieved good accuracies while further investigations showed considerable potential for going beyond this level of analysis to quantify size and number of secondary structure units.

The success of these hybrid approaches to protein structure predictions using experimental data should be predictable given the achievements of AI-driven tools such as AlphaFold and related platforms. This shows that, given sufficient information, the link between primary structure and 3D confirmation can be discerned. Our approach is similar, but with the focus on unravelling the spectrum–structure relationship. It is however of note that our ML methods do this successfully by using a very different portion of the spectrum to that which most previous 2D-IR studies have focused on. We thus believe that this study marks an encouraging step along the road to implementation of 2D-IR in applications ranging from quality control and regulation, to structure-based drug design involving dynamic protein structures.

## Author contributions

Amy Farmer: investigation, data curation, formal analysis, validation, visualization, writing original draft. Kelly Brown: investigation, validation, writing – review and editing. Sophie E. T. Kendall-Price: investigation, writing – review and editing. Partha Malakar: investigation, writing – review and editing. Gregory M. Greetham: funding acquisition, supervision, writing – review and editing. Neil T. Hunt: funding acquisition, methodology, validation, supervision, writing – review and editing.

## Conflicts of interest

There are no conflicts to declare.

## Supplementary Material

SC-017-D5SC09973K-s001

## Data Availability

Supplementary information (SI) is available. See DOI: https://doi.org/10.1039/d5sc09973k.
