# Supplementary material for: Dynamic protein structures in solution: decoding the amide I band with 2D-IR spectral libraries and machine learning
Source: Chem Sci. 2026 Jan 8;17(8):4285–95. doi: 10.1039/d5sc09973k (PMC12794349; doi:10.1039/d5sc09973k)
Supplement: SC-017-D5SC09973K-s001 [file SC-017-D5SC09973K-s001.pdf]

Please do not adjust margins

ARTICLE

# Dynamic Protein Structures in Solution: Decoding the Amide I Band with 2D-IR Spectral Libraries and Machine Learning

Amy L. Farmer,[a] Kelly Brown,[a] Sophie E.T. Kendall-Price,[a] Partha Malakar,[b] Gregory M. Greetham[b] and Neil T. Hunt*[a]

## Supporting Information

**Table S1**. The proportions of α-helix, parallel, antiparallel and total β-sheet calculated using the DSSP, the assigned structural and helix classes, and the number of α-helices of the proteins investigated.

| Protein (Abbr.) | α-helix content | Total β-sheet content | Structural Class | Helix Class | Number of α-helices | Parallel β-sheet content | Antiparallel β-sheet content |
|---|---|---|---|---|---|---|---|
| Myoglobin (Myo) | 0.7124 | 0 | 0 | 1 | 8 | 0 | 0 |
| HSA | 0.6861 | 0 | 0 | 1 | 29 | 0 | 0 |
| Calmodulin (Cal) | 0.5114 | 0.0238 | 0 | 0 | 8 | 0 | 0.0238 |
| Peroxidase (Per) | 0.4444 | 0.0196 | 0 | 0 | 13 | 0 | 0.0196 |
| Cytochrome c (Cyto) | 0.4135 | 0 | 0 | 1 | 5 | 0 | 0 |
| Glycogen phosphorylase. b (Gly b) | 0.4327 | 0.1419 | 0 | 1 | 33 | 0.0710 | 0.0698 |
| Lysozyme (Lys) | 0.3101 | 0.0620 | 0 | 0 | 4 | 0 | 0.0620 |
| Creatine (Cre) | 0.3526 | 0.1421 | 0 | 1 | 14 | 0 | 0.1395 |
| DT Diaphorase (DT) | 0.2894 | 0.1136 | 1 | 0 | 7 | 0.1136 | 0 |
| Lipoxidase (Lip) | 0.3048 | 0.1305 | 1 | 1 | 27 | 0 | 0.1198 |
| Lactoferrin Bovine (LB) | 0.2920 | 0.1748 | 1 | 1 | 21 | 0.0521 | 0.1200 |
| Protease (Pro) | 0.2956 | 0.1788 | 1 | 1 | 9 | 0.1314 | 0.0401 |

[a.] Department of Chemistry and York Biomedical Research Institute, University of York, York, UK
[b.] STFC Central Laser Facility, Research Complex at Harwell, Harwell Science and Innovation Campus, Didcot, UK
* Corresponding author email: neil.hunt@york.ac.uk
Supplementary Information available: [details of any supplementary information available should be included here]. See DOI: 10.1039/x0xx00000x

Please do not adjust margins

Please do not adjust margins



| Protein (Abbr.) | α-helix content | Total β-sheet content | Structural Class | Helix Class | Number of α-helices | Parallel β-sheet content | Antiparallel β-sheet content |
|---|---|---|---|---|---|---|---|
| Apo Transferrin (Apo) | 0.2960 | 0.1811 | 1 | 1 | 22 | 0.0574 | 0.1208 |
| Lactoferrin Human (LH) | 0.2967 | 0.1881 | 1 | 1 | 21 | 0.0550 | 0.1288 |
| Catalase (Cat) | 0.2725 | 0.1663 | 1 | 1 | 14 | 0 | 0.1632 |
| Conalbumin (Con) | 0.2656 | 0.1756 | 1 | 0 | 20 | 0.0566 | 0.1161 |
| α-amylase (amy) | 0.2337 | 0.1986 | 1 | 1 | 11 | 0.0579 | 0.1324 |
| Choline Oxidase (Chol) | 0.2086 | 0.2068 | 1 | 1 | 11 | 0.0733 | 0.1184 |
| Glyceraldehyde-3-phosphate dehydrogenase (Gly 3) | 0.2530 | 0.2651 | 1 | 1 | 8 | 0.1265 | 0.1295 |
| Ovalbumin (Ova) | 0.2902 | 0.3131 | 1 | 1 | 11 | 0.0484 | 0.2545 |
| β-Glucuronidase (b glu) | 0.1758 | 0.2968 | 1 | 1 | 8 | 0.0697 | 0.2073 |
| Ribonuclease A (RNase) | 0.1774 | 0.3306 | 1 | 0 | 3 | 0.0081 | 0.3065 |
| Ubiquitin (Ubi) | 0.1579 | 0.3158 | 1 | 0 | 1 | 0.0658 | 0.2500 |
| α-2-Macroglobulin (a-2) | 0.1245 | 0.2997 | 1 | 1 | 16 | 0.0178 | 0.2744 |
| Chymotrypsinogen A (Chym A) | 0.0735 | 0.3224 | 2 | 0 | 2 | 0.0041 | 0.2980 |
| Thaumatin (Thau) | 0.1063 | 0.3575 | 2 | 0 | 4 | 0.0290 | 0.2899 |
| Trypsin Chymotrypsin Inhibitor (TCI) | 0 | 0.2702 | 2 | 0 | 0 | 0 | 0.2702 |
| Elastase (Elas) | 0.0583 | 0.3417 | 2 | 0 | 2 | 0 | 0.3208 |
| β-Lactoglobulin (b lacto) | 0.0988 | 0.4074 | 2 | 0 | 2 | 0 | 0.3889 |
| Pepsin A (Pep) | 0.1104 | 0.4356 | 2 | 0 | 6 | 0.0583 | 0.3466 |
| Trypsin Inhibitor A (Tryp) | 0 | 0.3465 | 2 | 0 | 0 | 0.0057 | 0.3408 |
| Superoxide Dismutase (SD) | 0.0265 | 0.3907 | 2 | 0 | 1 | 0 | 0.3709 |
| IgG | 0.0323 | 0.4373 | 2 | 0 | 4 | 0.0210 | 0.4020 |
| Prealbumin (Pre) | 0.0472 | 0.4803 | 2 | 0 | 1 | 0.1102 | 0.3543 |
| Concanavalin A (Con A) | 0 | 0.4336 | 2 | 0 | 0 | 0 | 0.4304 |


This journal is © The Royal Society of Chemistry 20xx

Please do not adjust margins

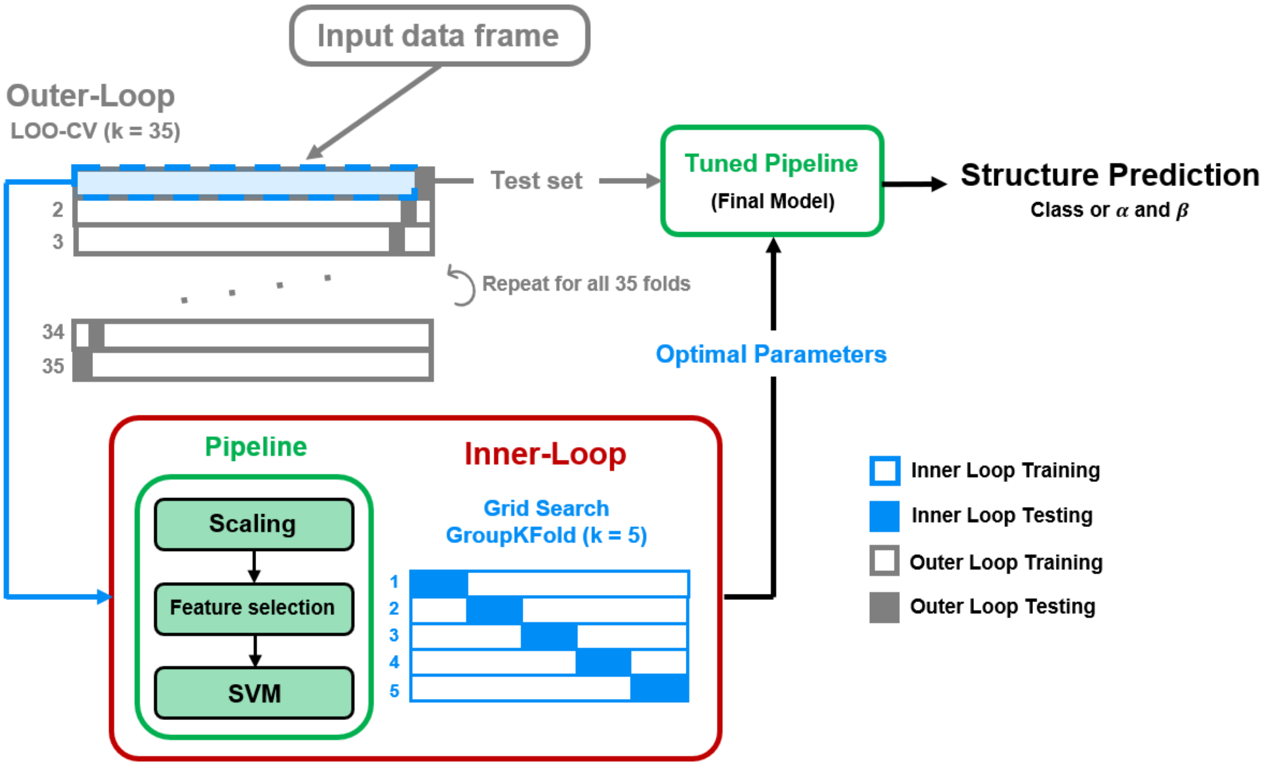


**Figure S1**. A schematic diagram describing the ML analysis framework. The input data frame is first passed through an outer-loop. A Leave-One-Out (LOO) has been represented here, where all of the spectra of each protein are removed iteratively for use as a final test set to evaluate model performance. The training set of each iteration of the outer-loop is then passed through an inner-loop cross-validation (CV) where a hyperparameter space is searched using a pipeline of two data transformers (scaling and feature selection) and a final predictor (SVM in the example here) across a group Grid Search CV. This identifies a set of optimal parameters and number of features selected to produce a final tuned pipeline. This tuned pipeline then performs a structure prediction for the test set of the given outer-loop iteration.

Please do not adjust margins



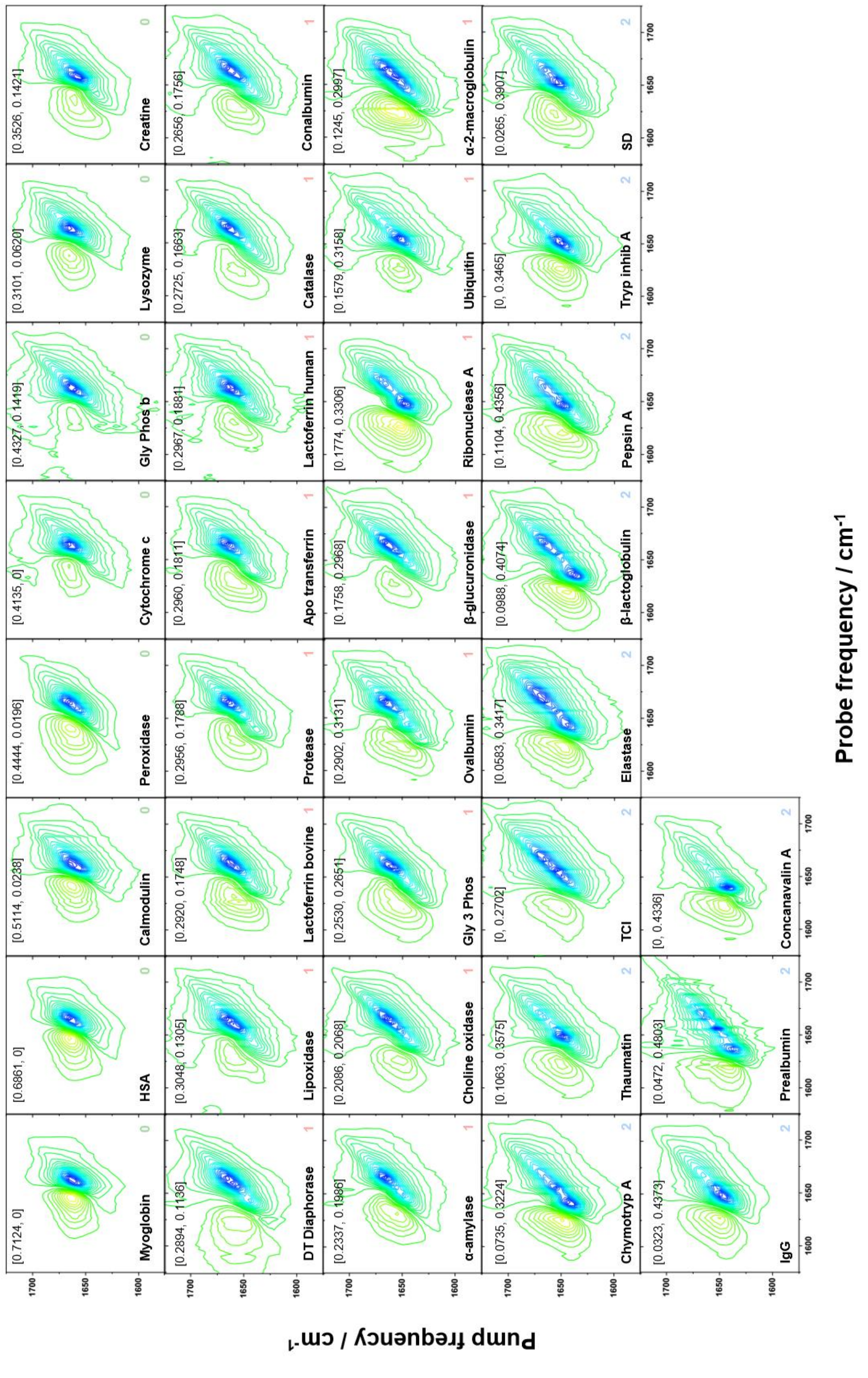


**Figure S2**. Example Amide I 2D-IR spectra of the 35 proteins in the protein library. The numbers in the square brackets refer to the proportions of α-helix and β-sheet determined through the DSSP [α,β], and the numbers in the bottom right corner of each spectrum indicate the structural class assignment of the protein.



This journal is © The Royal Society of Chemistry 20xx

Please do not adjust margins

Please do not adjust margins



**Table S2**. The average F1 scores across three randomly generated test sets (80:20 training: testing split) for the 8 methods tested when trained on the diagonal or 2D library.

| | Diagonals | | | 2D | | |
|---|---|---|---|---|---|---|
| **Method** | ***α-enriched*** | ***Mixed structures*** | ***β-enriched*** | ***α-enriched*** | ***Mixed structures*** | ***β-enriched*** |
| PCA-SVC | 0.73 | 0.90 | 0.71 | 0.50 | 0.75 | 0.42 |
| PCA-kNN | 0.57 | 0.76 | 0.51 | 0.54 | 0.65 | 0.38 |
| PCA-DT | 0.47 | 0.72 | 0.52 | 0.48 | 0.50 | 0.44 |
| PCA-RF | 0.57 | 0.78 | 0.45 | 0.49 | 0.69 | 0.42 |
| AF-SVC | 0.62 | 0.86 | 0.58 | 0.80 | 0.90 | 0.90 |
| AF-kNN | 0.80 | 0.86 | 0.58 | 0.73 | 0.87 | 0.84 |
| AF-DT | 0.72 | 0.83 | 0.71 | 0.67 | 0.84 | 0.85 |
| AF-RF | 0.62 | 0.81 | 0.41 | 0.62 | 0.85 | 0.83 |

This journal is © The Royal Society of Chemistry 20xx



Please do not adjust margins

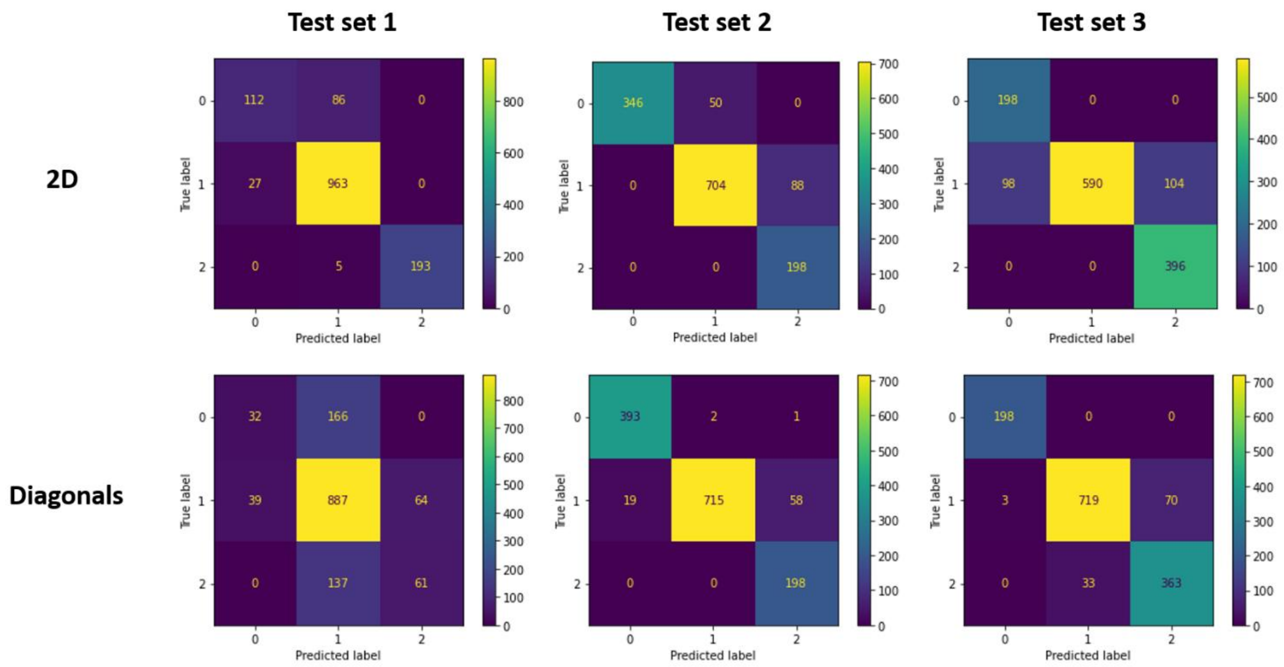


**Figure S3**. The confusion matrices for the three test sets of the outer-loop CV of three 80:20 (training:testing) splits using the AF-SVC model trained on the 2D library (top row) and the the PCA-SVC model trained on the diagonals library (bottom row). Class 0, 1 and 2 refer to the α-enriched, mixed structures and β-enriched classes, respectively. In the confusion matrices, numbers along the diagonal represent correct classifications, whilst numbers in the off-diagonal boxes represent incorrect classifications.


This journal is © The Royal Society of Chemistry 20xx

Please do not adjust margins



**Table S3**. The training accuracies, testing accuracies, κ-values, precision values, recall values, and F1 scores for the three test sets of the outer-loop CV of three 80:20 (training: testing) splits using the AF-SVC model trained on the 2D library, and the PCA-SVC model trained on the diagonal library.

| Library | Test set | Training Accuracy / % | Testing Accuracy / % | $\kappa$ | Class | Precision | Recall | F1 score |
|---|---|---|---|---|---|---|---|---|
| 2D | 1 | 86 ± 16 | 91.5 | 0.80 | 0 | 0.806 | 0.566 | 0.665 |
| | | | | | 1 | 0.914 | 0.973 | 0.942 |
| | | | | | 2 | 1 | 0.975 | 0.987 |
| 2D | 2 | 82 ± 17 | 90.0 | 0.83 | 0 | 1 | 0.874 | 0.933 |
| | | | | | 1 | 0.934 | 0.889 | 0.911 |
| | | | | | 2 | 0.692 | 1 | 0.818 |
| 2D | 3 | 81 ± 22 | 85.4 | 0.77 | 0 | 0.669 | 1 | 0.802 |
| | | | | | 1 | 1 | 0.745 | 0.854 |
| | | | | | 2 | 0.792 | 1 | 0.884 |
| Diagonals | 1 | 81 ± 16 | 70.7 | 0.20 | 0 | 0.346 | 0.045 | 0.275 |
| | | | | | 1 | 0.714 | 0.879 | 0.197 |
| | | | | | 2 | 0.275 | 0.788 | 0.229 |
| Diagonals | 2 | 73 ± 20 | 94.2 | 0.90 | 0 | 0.954 | 0.992 | 0.973 |
| | | | | | 1 | 0.997 | 0.903 | 0.948 |
| | | | | | 2 | 0.770 | 1 | 0.870 |
| Diagonals | 3 | 85 ± 14 | 92.4 | 0.87 | 0 | 0.985 | 1 | 0.992 |
| | | | | | 1 | 0.956 | 0.908 | 0.931 |
| | | | | | 2 | 0.838 | 0.917 | 0.876 |



Please do not adjust margins

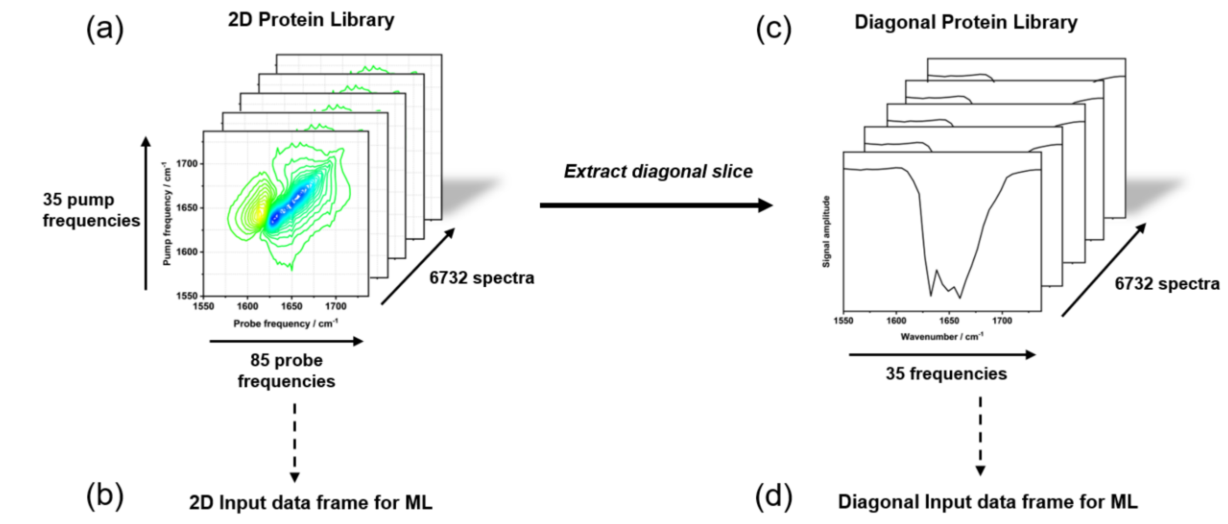


| | 1 | 2 | · · · · | 2975 |
|---|---|---|---|---|
| 1 | $A_{v_{pu,1},v_{pr,1}}$ | $A_{v_{pu,1},v_{pr,2}}$ | · · · · | $A_{v_{pu,35},v_{pr,85}}$ |
| 2 | $A_{v_{pu,1},v_{pr,1}}$ | $S_{v_{pu,1},v_{pr,2}}$ | · · · · | $A_{v_{pu,35},v_{pr,85}}$ |
| ⋮ | ⋮ | ⋮ | ⋱ | ⋮ |
| 6732 | $A_{v_{pu,1},v_{pr,1}}$ | $A_{v_{pu,1},v_{pr,2}}$ | · · · · | $A_{v_{pu,35},v_{pr,85}}$ |

| | 1 | 2 | · · · · | 35 |
|---|---|---|---|---|
| 1 | $A_{v_1}$ | $A_{v_2}$ | · · · · | $A_{v_{35}}$ |
| 2 | $A_{v_1}$ | $A_{v_2}$ | · · · · | $A_{v_{35}}$ |
| ⋮ | ⋮ | ⋮ | ⋱ | ⋮ |
| 6732 | $A_{v_1}$ | $A_{v_2}$ | · · · · | $A_{v_{35}}$ |

**Figure S4**. Schematic diagram outlining how the 2D-IR protein library (a) was formatted into a 2D input data frame for ML analysis (b), and converted into a diagonal protein library (c), with a similar input data frame for ML analysis (d).


This journal is © The Royal Society of Chemistry 20xx

Please do not adjust margins



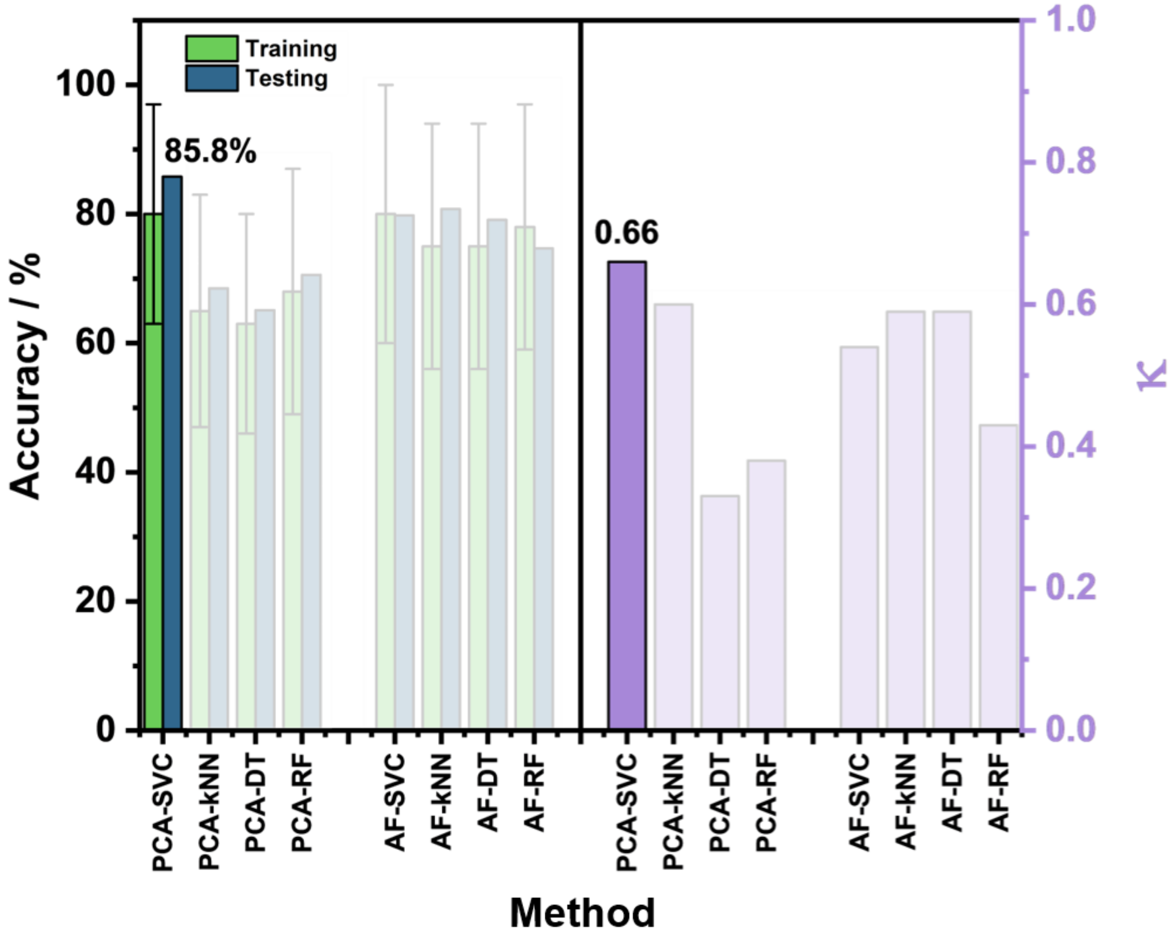


**Figure S5**. (Left panel) The average training accuracies across a 10-fold cross-validation and testing accuracies, and (Right panel) Cohen's kappa values for the 8 methods trained on the diagonal library. The best performing method, PCA-SVC, has been highlighted in both panels.



Please do not adjust margins

Please do not adjust margins



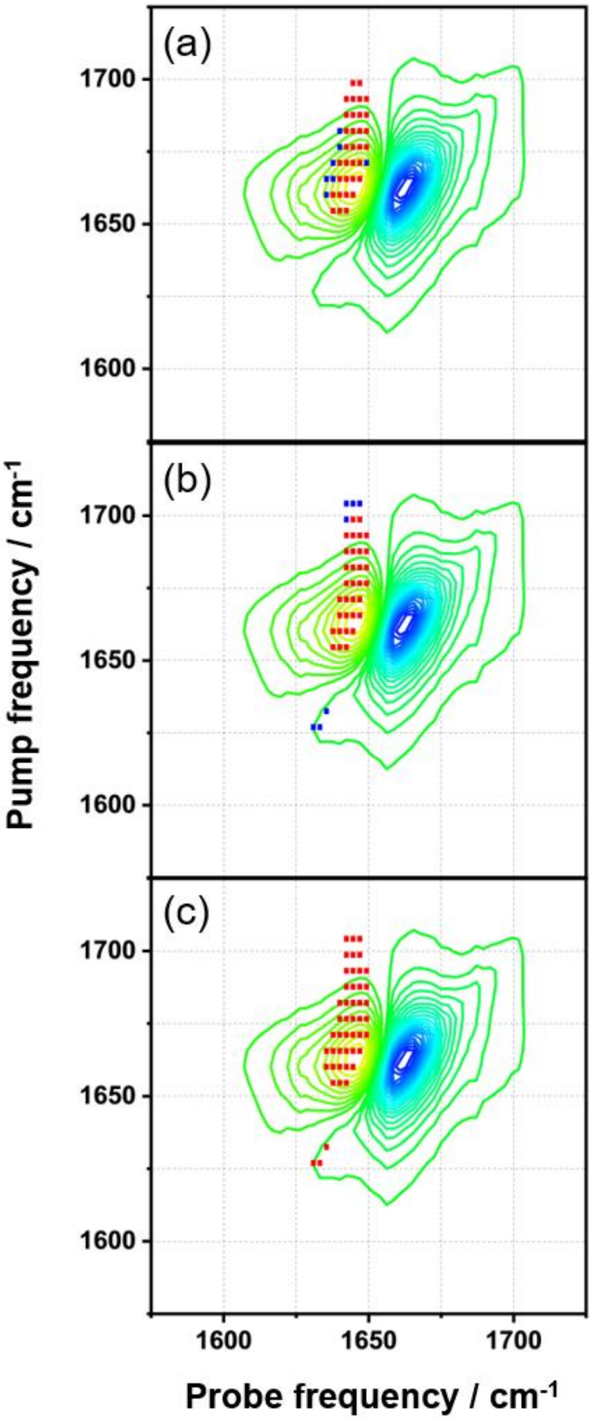


**Figure S6**. The positions of the 40 features with the highest F-values from an ANOVA-F test on the total protein library labelled with (a) the proportions of α-helix and (b) the proportions of β-sheet overlaid with the 2D-IR spectrum of Myoglobin. The blue squares represent features uniquely selected by each F-test whilst the red squared represent the features selected by both tests. (c) The combined features from (a) and (b).



This journal is © The Royal Society of Chemistry 20xx

Please do not adjust margins

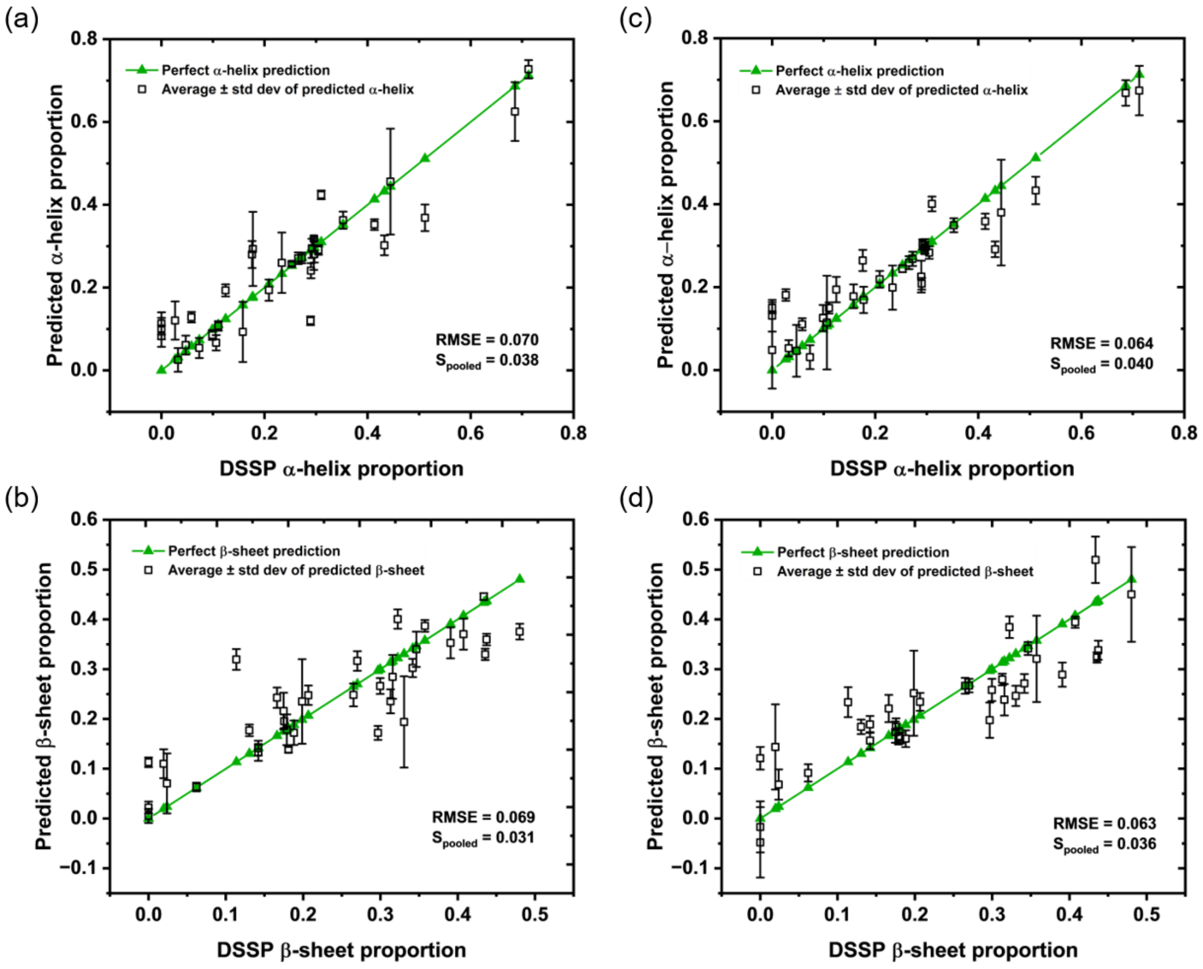


**Figure S7**. The predicted (a) α-helix and (b) β-sheet proportions from a Leave-One-Out (LOO) analysis using the AF-SVR pipeline trained on the 2D library, where each open black square represents the average predicted proportion across the repeat spectra in each protein group. (c) and (d) show the predicted α-helix and β-sheet proportions, respectively, from the LOO analysis using the PCA-SVR model trained on the diagonal library. In each panel, the green line represents a perfect prediction where the green triangles are positioned at the DSSP calculated proportions of α-helix and β-sheet for the 35 proteins.

Please do not adjust margins



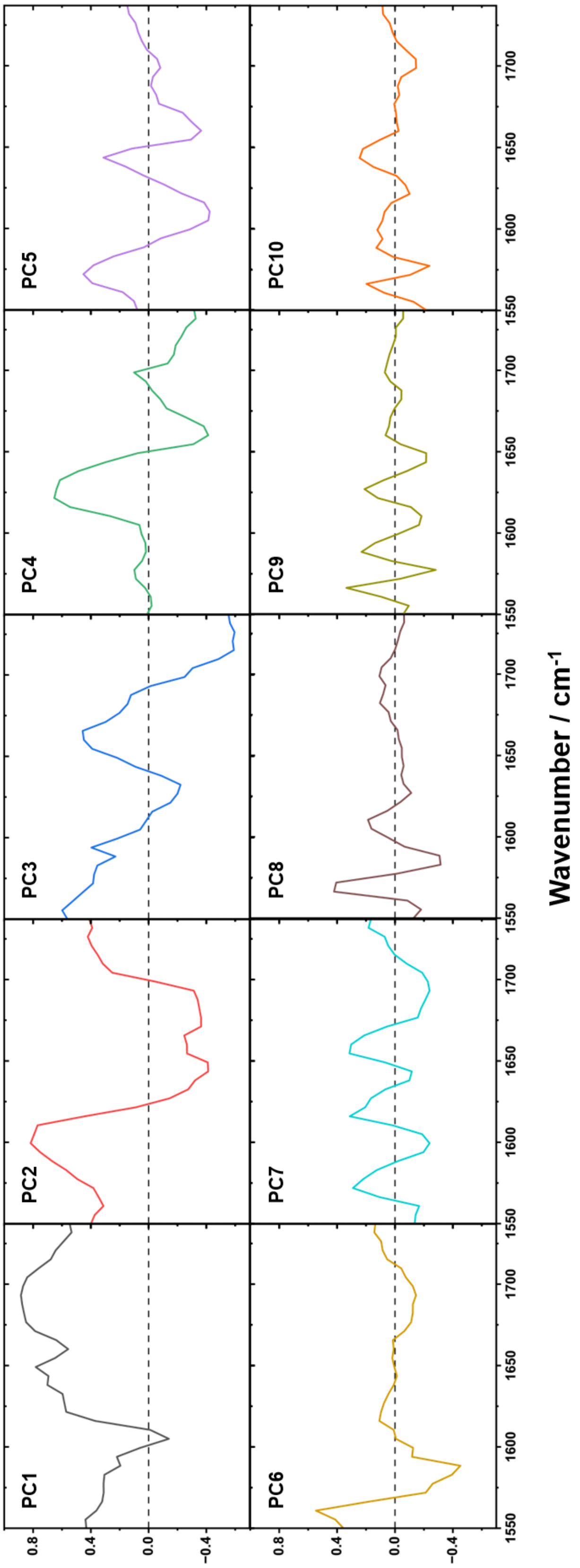


**Figure S8**. The first 10 principal components (PCs) used in the PCA-SVC model trained on the diagonal library.



This journal is © The Royal Society of Chemistry 20xx

Please do not adjust margins

Please do not adjust margins



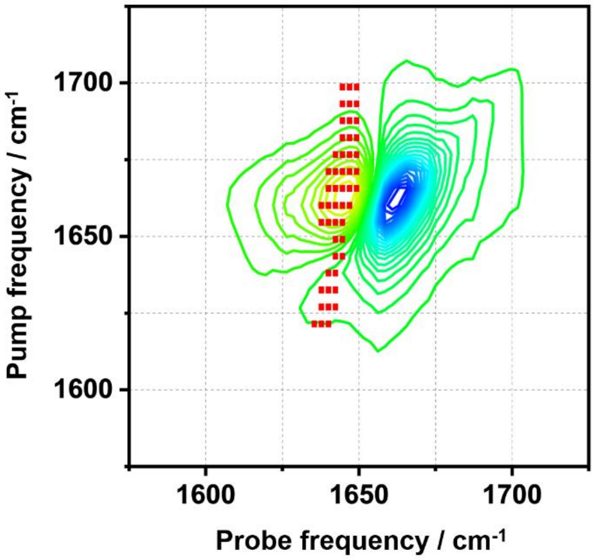


**Figure S9.** The 50 highest scoring features from an ANOVA-F test on the total 2D protein library with the helix class label as the target variable overlaid with the 2D-IR spectrum of Myoglobin.



Please do not adjust margins

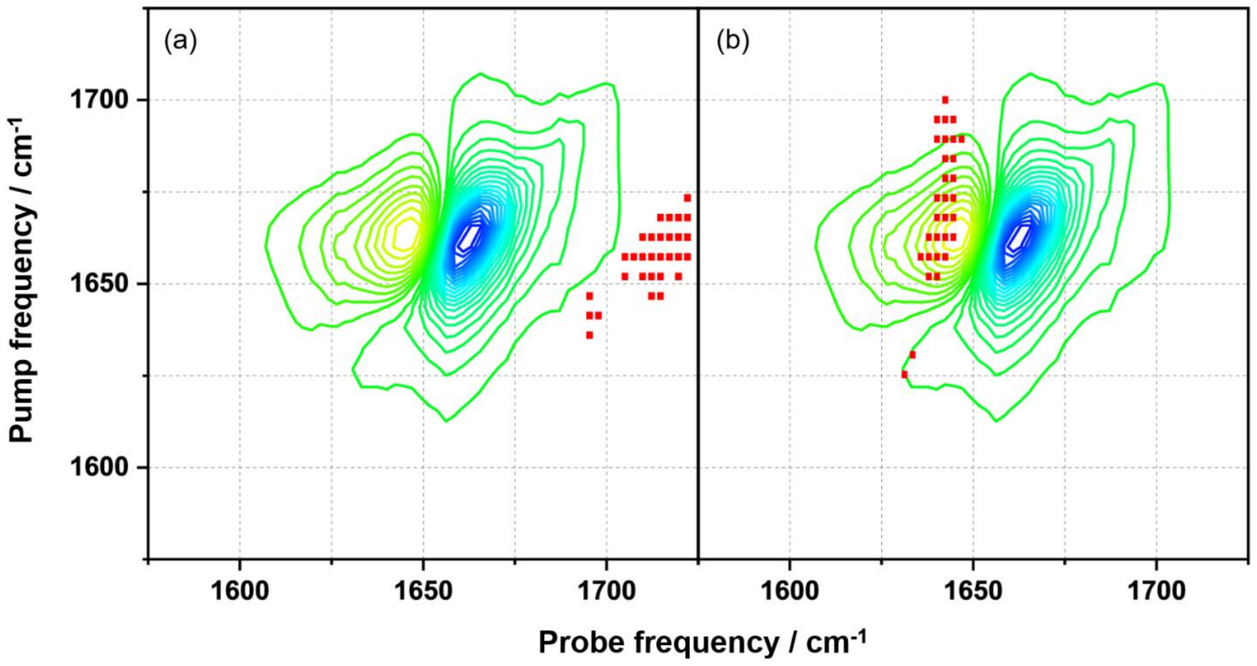


**Figure S10.** The selected features from an ANOVA-F test on the total 2D library with (a) the proportions of parallel β-sheet as the target variable, and (b) the proportions of antiparallel β-sheet as the target variable. Both sets of features have been overlaid with the 2D-IR spectrum of Myoglobin.


This journal is © The Royal Society of Chemistry 20xx

Please do not adjust margins



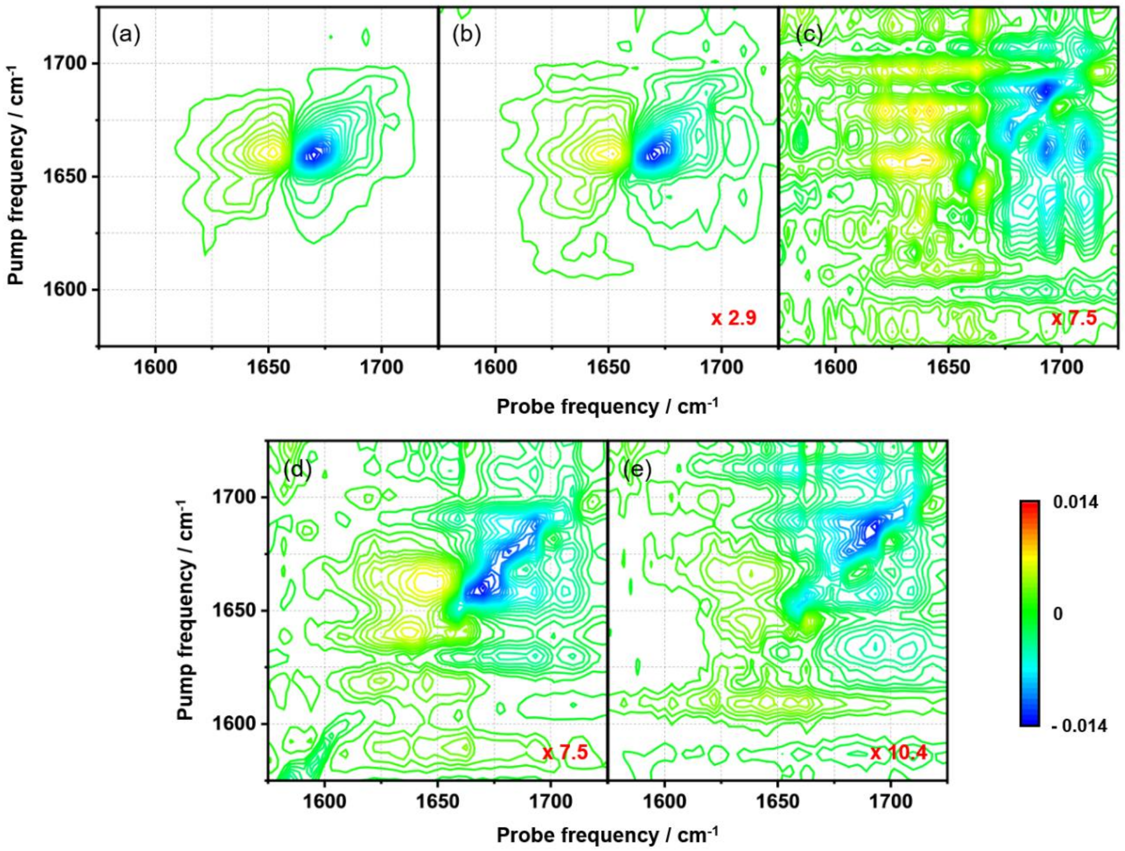


**Figure S11.** (Top row) The 2D-IR spectra of Bovine Serum Albumin (BSA) in $H_2O$ at (a) 45 mg/mL and (b) 15 mg/mL (c) Is the difference spectrum between 15 mg/mL and 45 mg/mL where the 15 mg/mL spectrum has been scaled to the 45 mg/mL spectrum at the position of minimum intensity of the bleach. In this difference spectrum, there is no identifiable concentration effect. Only following magnification is any response clear, but this is mostly noise differences and there is no residual protein signal. (Bottom row) The 2D-IR spectra of (d) BSA in $H_2O$ at 5 mg/mL and (e) the $H_2O$-based phosphate buffer used to prepare the BSA solution. At 5 mg/mL, the protein signal is still visible (~ 1660 $cm^{-1}$), but the spectrum is influenced by a significant $H_2O$ response from the phosphate buffer (e). This is also apparent in the difference spectrum (c). The red numbers in each panel are the magnification factors used to set the spectra to the same z-axis.

For Figure S11, the data were collected using different instrumentation to that used to collect the protein library data. The details of the 2D-IR spectroscopy for Fig. S11 are given below.

The 2D-IR spectrometer comprised two Yb-based amplified lasers (Pharos 20W and Pharos 10W, Light Conversion) synchronised by a common oscillator. Each amplifier was used to pump an optical parametric amplifier (OPA, Orpheus Mid-IR, Light Conversion) equipped with difference frequency generation to produce independently tuneable pump and probe sources for one or two-colour 2D-IR spectroscopy. The OPAs produced bandwidths of > 200 $cm^{-1}$ with pulse energies of 2.5 and 1.5 μJ, respectively, at a pulse repetition rate of 50 kHz. 2D-IR data were collected via a 2DQuick spectrometer (Phasetech) that used pump-probe beam geometry and a mid-IR pulse shaper to generate and control the time delay (τ) between the pair of pump pulses. The signal was detected using a 64-element HgCdTe array detector. Each sample was measured at ZZZZ (parallel) polarization geometry and a waiting time ($T_w$) of 250 fs. For the given $T_w$, τ was scanned in steps of 24 fs to a maximum delay of 3 ps, applying a rotating frame frequency of 1208 $cm^{-1}$. Each 2D-IR spectrum represents the average of 1000 spectra, repeated 3 times.



Please do not adjust margins
